# Coding choices and short-sequence artifacts undermine claims of a conventional Paleolithic sign system

**DOI:** 10.1073/pnas.2627836123

**Published:** 2026-09-11

**Authors:** Alexander Koplenig

**Affiliations:** ^a^ Department of Lexical Studies, Leibniz Institute for the German Language, Mannheim 68161, Germany

Bentz and Dutkiewicz (1, B&D) conclude that 40,000-y-old Aurignacian signs were used in a deliberate, systematic, and conventional manner. I identify four problems in their analyses.

B&D characterize each sequence by four features: unigram entropy (*H*), entropy rate (*h*), type-token ratio, and repetition rate. First, B&D’s Fig. 1A illustrates two coding problems: spaces and underscores—classified in their data as annotations rather than signs—enter the calculations as signs, while visually differing engraved marks are coded as one type (“°”). Whether Paleolithic people regarded them—within and across objects—as one type or several cannot be established; B&D acknowledge elsewhere that sign typology is partly subjective (2), yet do not account for this uncertainty. Ten independent AI visual classifications identified multiple types. Removing annotations or using these alternative visual codings changes all four features for this object; annotation removal also shifts all four across the Aurignacian corpus (Fig. 1 *A* and *B*). After removing annotations, 143 of 213 Aurignacian strings (67%) repeat a single sign type.

**Fig. 1.**
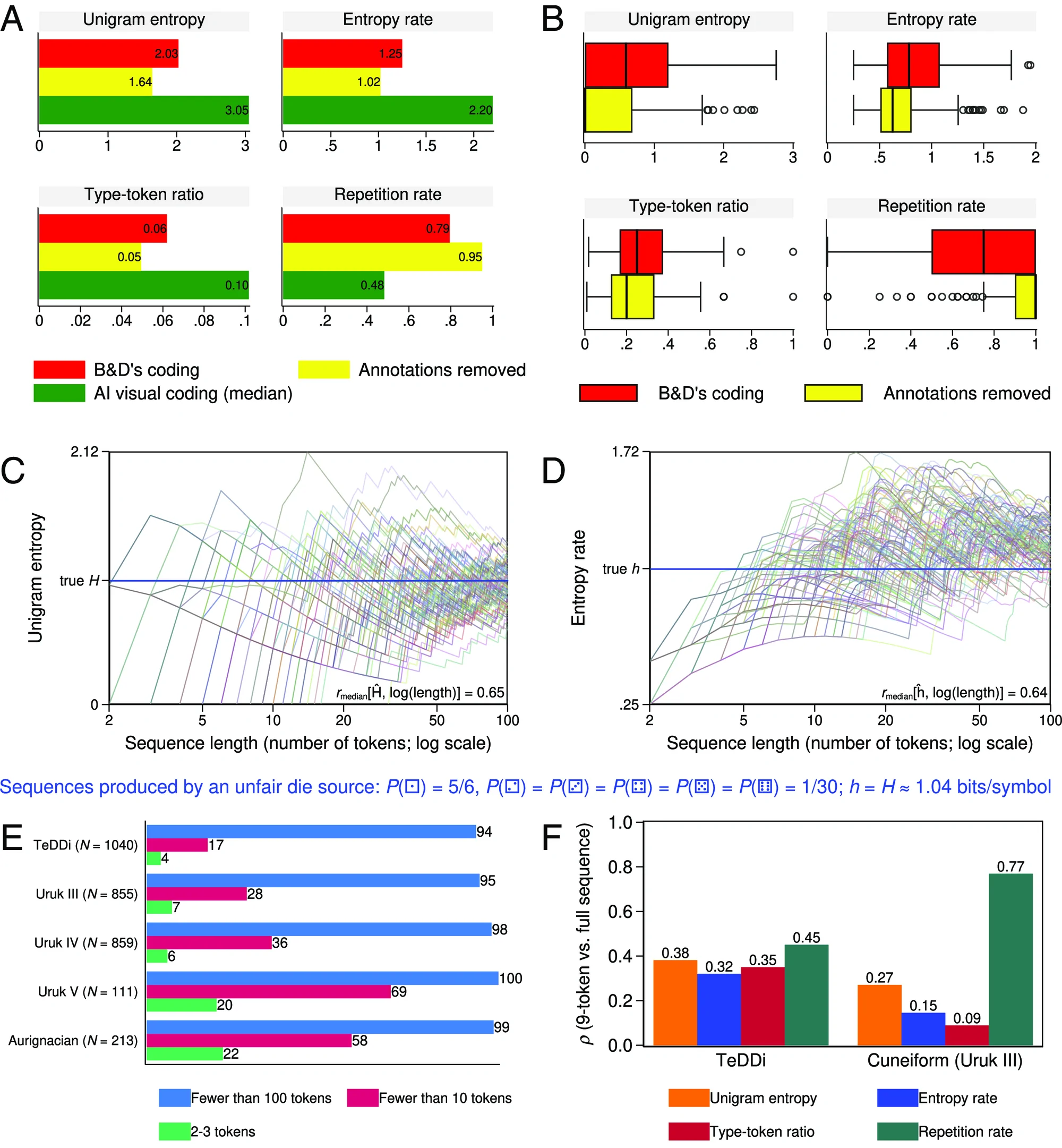
(*A*) Four statistical features for object *gkl0025* under B&D’s original coding, after removing spaces and underscores, and as medians across 10 independent AI visual classifications; prompt and outputs at https://osf.io/cuf3z (3). (*B*) Distributions of the same four features across all 213 Aurignacian sequences before and after removing spaces and underscores. (*C* and *D*) Estimated unigram entropy and entropy rate as functions of token count for 100 sequences generated by the unfair-die source specified below the panels. (*E*) Percentages of sequences containing fewer than 100 tokens, fewer than 10 tokens, and two or three tokens across subcorpora; sample sizes in parentheses. (*F*) Spearman correlations between nine-token and full-sequence estimates of all four features for TeDDi (*N* = 66) and Uruk III (*N* = 46), restricted to sequences containing at least 100 tokens.

Second, B&D describe information density as “a fundamental property of a sign sequence.” This is misleading: *H* and *h* characterize the probabilistic source generating sequences—*H* the uncertainty over sign types, *h* the unpredictability of successive tokens given preceding ones; an observed sequence is only a sample from which both are estimated (4–7). Inferring *h* from two or three tokens is analogous to inferring an unfair die’s probabilities from two or three throws. With an unfair die, *H* and *h* estimates vary strongly at short sequence lengths despite known true values (Fig. 1 *C* and *D*). Yet >95% of B&D’s 3,078 sequences contain fewer than 100 tokens, >30% fewer than 10, and 220 only two or three (Fig. 1*E*). Studies commonly analyze thousands to millions of tokens using these estimators (7–10). Nine-token estimates from protocuneiform and modern written sequences likewise often correspond poorly with full-sequence estimates (Fig. 1*F*).

Third, I show that sequence length and the number of different signs systematically influence the classification results: Random within-corpus splits produce no above-baseline classification, whereas their classifier readily distinguishes shorter from longer sequences and type counts alone reproduce the published cross-corpus pattern (Fig. 2 *A*–*D*). Aurignacian and Uruk V are the only subcorpus-pair not differing significantly in tokens or types (Fig. 2*E*). Their apparent statistical similarity is therefore likely to reflect their similarly short sequences and low type counts relative to other corpora, rather than a shared “statistical fingerprint.” Indeed, replacing the Aurignacian sequences with length-matched random dice sequences reproduces the classification pattern (Fig. 2 *F* and *G*).

**Fig. 2.**
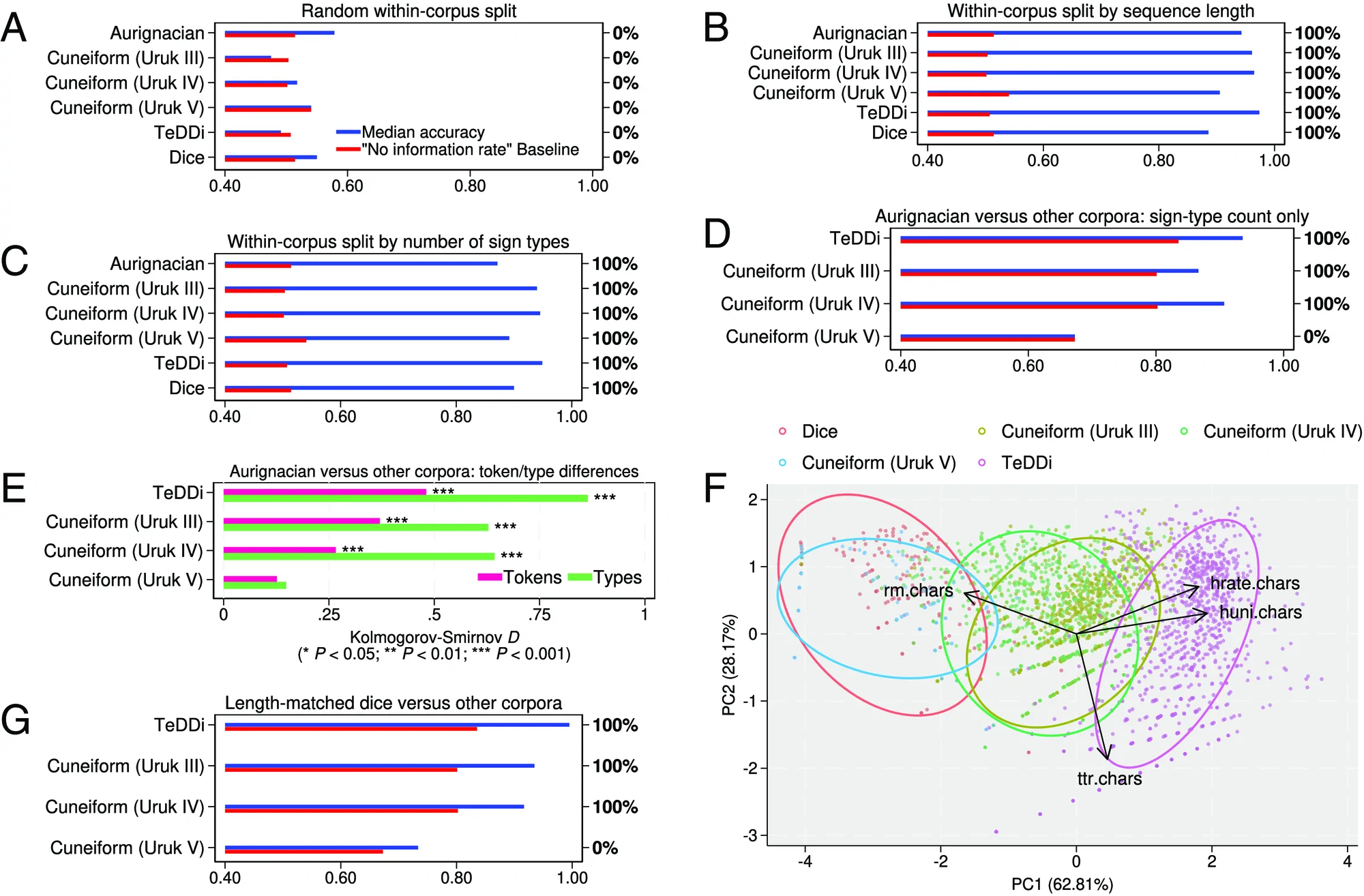
(*A*) KNN classification results for random within-corpus splits. (*B*) KNN results for lower vs. upper within-corpus halves by token count. (*C*) KNN results for lower vs. upper within-corpus halves by type count. (*D*) Pairwise classification of Aurignacian against other subcorpora using type count only (compare B&D’s SI Appendix, figure S4 C and D). (*E*) Kolmogorov–Smirnov distances between Aurignacian and other subcorpora for token- and type-count distributions; larger values indicate greater differences. (*F*) Principal component analysis of B&D’s four features after replacing Aurignacian with the matched dice corpus (compare B&D’s figure 3D). (*G*) Pairwise KNN classification of the dice corpus against other subcorpora (compare B&D’s SI Appendix, figure S4 C and D). In (*A*–*D* and *G*), right-hand numbers give the percentage of *k* = 1, …, 10 values with accuracy significantly above baseline (one-sided, Bonferroni-adjusted *P* < 0.05; see B&D, “Classification of Sign Sequences”). Panels *A*–*C* and *G* use B&D’s four features. Dice sequences in *F* and *G* match Aurignacian sequence lengths and comprise independent throws from the unfair-die source in Fig. 1 *C* and *D*.

Finally, B&D’s figurine result is likewise not robust. Adding log-transformed token and type counts as covariates greatly improves B&D’s selected model (*AIC*: −249 vs. 144; *R*^2^: 89.51% vs. 27.96%). Only personal ornaments then have lower estimated *h* than tools (*P* = 0.007); both figurine-category estimates are negative but insignificant (*P*s > 0.2).

These problems affect every stage, from data encoding and statistical estimation to classification and interpretation. The reanalyses therefore cast serious doubt on whether the findings demonstrate a conventional Paleolithic sign system or information density comparable to protocuneiform.

## Data Availability

Code and data needed to reproduce the results are available at https://osf.io/cuf3z (3). I used the data made available by ref. 1.
